# Methodological Aspects of Multiplex Terminal Restriction Fragment Length Polymorphism-Technique to Describe the Genetic Diversity of Soil Bacteria, Archaea and Fungi

**DOI:** 10.3390/s20113292

**Published:** 2020-06-09

**Authors:** Agata Gryta, Magdalena Frąc

**Affiliations:** Institute of Agrophysics, Polish Academy of Sciences, Doświadczalna 4, 20-290 Lublin, Poland; m.frac@ipan.lublin.pl

**Keywords:** microbial genetic diversity, soil microbiome, genetic fingerprinting, capillary electrophoresis, multiplex t-RFLP

## Abstract

The molecular fingerprinting methods used to evaluate soil microbial diversity could also be used as effective biosensors for the purposes of monitoring ecological soil status. The biodiversity of microorganisms is a relevant index of soil activity and there is a necessity to develop tools to generate reliable results for an emerging approach in the field of environmental control using microbial diversity biosensors. This work reports a method under development for determining soil microbial diversity using high efficiency Multiplex PCR-Terminal Restriction Fragment Length Polymorphism (M-T-RFLP) for the simultaneous detection of bacteria, archaea and fungi. Three different primer sets were used in the reaction and the analytical conditions were optimized. Optimal analytical conditions were achieved using 0.5 µM of primer for bacteria and 1 µM for archaea and fungi, 4 ng of soil DNA template, and HaeIII restriction enzyme. Comparative tests using the proposed analytical approach and a single analysis of each microorganism group were carried out to indicate that both genetic profiles were similar. The Jaccard similarity coefficient between single and multiplexing approach ranged from 0.773 to 0.850 for bacteria and fungi, and 0.208 to 0.905 for archaea. In conclusion, the multiplexing and pooling approaches significantly reduced the costs and time required to perform the analyses, while maintaining a proper effectiveness.

## 1. Introduction

Communities of soil microorganisms play a crucial role in processes that are fundamental for life on Earth, causing the circulation of elements and facilitating plant growth [1,2]. Soil microbial communities vary across time and space, and are responsive to various factors, such as climate change, land management and pollution. Soil is therefore one of the most diverse ecosystems on our planet, [3,4]. The proper functioning of the soil ecosystem depends mainly on microbial metabolism and the ability of soil microbiome responses to change with the conditions in the environment. The diversity of bacteria, fungi and archaea determines the physiological status of the soil. Moreover, genetic diversity indices may be effective bioindicators of changes occurring in the soil. Knowledge concerning the soil microbiome is essential for maintaining high soil quality and health [5]. Therefore, it is necessary to find and elaborate upon an efficient and effective method using biosensors for the evaluation of the soil microbiome. Microorganisms are living and sensitive elements of soil, and are capable of reacting rapidly and detectably to various conditions and changes in the soil environment. The involvement of soil microorganisms, directly or indirectly, with each type of soil transformation makes them a reliable indicator of, and factor for, biosensors.

### 1.1. Development of Culture-Independent Methods to Describe Microbial Communities

In recent decades, culture-independent methods to describe communities of microorganisms have been developed [6]. These approaches are based on an analysis of DNA extracted from environmental samples, excluding the steps of isolation and the culturing of microorganisms, and therefore they could be an important, fast and reliable tool in the field of biosensors for environmental control based on microbial diversity. Despite the number of molecular methods that are well described and have been applied to characterize the soil microbiome, there are still gaps in our knowledge concerning the comprehension of the ecological process [7,8,9]. Several previous studies have indicated that multi-taxonomic approaches are more reliable for the assessment of the ecological status of soil [10,11]. The soil microbiome consists of bacteria, archaea and fungi, and it is obvious that the biotic interactions between these components are highly important in determining ecosystem processes. Therefore, a multi-taxonomic biodiversity-based biosensing approach may be a good alternative to single microbial group-based biodiversity sensors since it can offer the benefits of lower cost and usability on a wide range of targets in one reaction.

Culture-independent methods based on studying microbial communities, such as fingerprinting techniques (Denaturing Gradient Gel Electrophoresis (DGGE), Terminal Restriction Fragment Length Polymorphism (t-RFLP), or next generation sequencing (NGS)) are important sources of information about the composition of microbial communities [12]. These methods are the techniques most frequently used to describe changes in microbial community structure and diversity [13]. Through the use of appropriately selected primers, specific for individual taxons, it is possible to characterize a few microbial groups in the same DNA sample through the use of individual PCR amplification. However, this practice is costly and time-consuming, especially in very extensive and accurate experiments. 

Multiplex t-RFLP is a culture-independent method used to describe and recognize multiple biomarkers of microbial communities [14], but may also be used as a microbial diagnostic tool [15,16,17]. In comparison with NGS, the t-RFLP approach remains a necessary tool to describe microbial diversity in ecosystems. Although the development of NGS provides the opportunity to achieve relevant results focused on species identification and detection of changes in environmental microbial composition, there remains the necessity to use a fingerprinting method such as t-RFLP to achieve a quick “snapshot” [18] of the microbiome. This could be an informative source of information about the differences and similarities in soil microbial communities in response to various land management or fertilization strategies, although it cannot be used as a tool for the identification of a key species in microbial communities. Furthermore, fingerprinting methods (t-RFLP) are rapid and much cheaper than NGS, and additionally provide highly reproducible results. In addition, a number of investigations have demonstrated consistent results with regard to community structures generated from the fingerprinting technique and NGS [19,20,21]. 

### 1.2. Microbial Community Analysis Based on t-RFLP

The application of the multiplex PCR-t-TRFLP approach allows a fast and effective use of biosensors to evaluate the genetic diversity of groups of microorganisms of interest in the soil microbial community. In brief, t-RFLP analysis is a sensitive fingerprinting biosensing method based on separating the different fragment lengths of DNA using electrophoresis. In the first step of t-RFLP, amplification is conducted with fluorescently labelled primers. Subsequently, restriction digestion and analysis are used to obtain the terminal restriction fragments with a genetic analyzer. Each sequence is characterized by a set of restriction fragments, which may be visualized with capillary electrophoresis, and the electropherogram produced is a pattern of peaks with different sizes. The fragments are determined by the height of the peak and their intensity (relative fluorescence). The obtained set of restriction fragments is characteristic and unique for tested communities, and creates specific pattern fingerprints. The terminal restriction fragments (t-RFs) may be assigned to databases and identified using specific tools such as a functional gene pipeline and repository [22] and TRiFLe software [23]. Therefore, they can be used as biosensors for describing microbial communities present in the soil environment.

The multiplex assay is a strategy to simultaneously analyze a large number of samples in one reaction, thus, the cost and time of study performing may be reduced. Multiplexing makes it possible to perform one common reaction for various groups of microorganisms, genus or species in a condition, when the parameters of the reaction will be appropriate for each tested element. The multiplexing might be applied to PCR and each next step is then performed with a mixture of multiplex PCR products. Another way to multiplex is to carry out the single reactions (PCR and restriction) and then perform multiplex fragment analysis of pooled samples. In both approaches, product fluorescent labeling is necessary to distinguish between them. 

The goal of the conducted study was to apply the traditional approach of the t-RFLP method to simultaneously analyze a number of microbial taxons with one reaction in agricultural soil samples. The main aim of the study was to report the progress in developing an effective multiplex t-RFLP approach for analysis of various microbial groups taking into account two different soil types. Additionally, this paper recommends ways to adjust conditions of the subsequent stages to obtain the best results for the soil samples. The research methodology described in detail in this paper is comprises a combination of literature research reports and our own adaptations and modifications that were necessary to optimize the best multiplex t-RFLP conditions, including pooling and multiplexing approaches.

## 2. Materials and Methods

Soil samples were collected in three replications from a field experiment with biofertilizer additions under corn cultivation, from two different soil types: Brunic Arenosol (S1) and Abruptic Luvisol (S2) with various physicochemical parameters: pH_KCl_ 4.8 and 4.9, content of P_2_O_5_ 17.4 and 4.8 mg 100 g^−1^, K_2_O 2.9 and 5.3 mg 100 g^−1^, Mg 1.2 and 3.6 mg 100 g^−1^, N-NH_4_ 2.14 and 6.57 mg kg^−1^ d.m., N-NO_3_ < 1.39 and 2.91 mg kg^−1^ d.m., high content of sand and silt for S1 and S2, respectively. The soil samples were delivered to the laboratory immediately after sampling, and the soil was sieved using a 2-mm sieve to eliminate plant fragments, stones and other impurities.

The DNA to be analyzed (in three replications) were extracted from 0.5 g of the soil sample using FastDNA^®^ SPIN Kit for Feces (MP Biomedicals, Solon, OH, USA) following the protocol supplied by the manufacturer. The amount and purity of the DNA was determined by a spectrophotometer (NanoDrop 2000/2000c Thermo Scientific, West Palm Beach, FL, USA) at 260/280 nm.

In order to perform PCR, three literature pairs of primers specific for bacteria, archaea and fungi (Table 1) were selected. The selected target region of amplification for bacteria and archaea was the 16S rDNA gene, a universal fragment for eubacteria/archaea, while for fungi, the target tested region included the internal transcribed spacer (ITS1) recommended as the universal and specific fungal barcode sequence.

Three t-RFLP approaches were applied for the analysis of three microbial groups (bacteria, archaea and fungi). In the single t-RFLP approach, each stage of analyses was performed separately for each group of microorganisms. The multiplex methods included pooling and multiplexing approaches are explained below. The pooling approach included individual PCR stage and common restriction digestion reaction for the mixture of PCR products (bacteria, fungi and archaea) and then common fragment analysis from mixture of digestion. Summarizing the multiplexing approach, each stage, starting from PCR and ending with fragment analysis, was performed simultaneously for each groups of microorganisms. An outline of the procedure performed is presented in Table 2.

The primary conditions of choice were the annealing temperature and the generally positive efficiency in the amplification tests. In the first step of optimization, the quality of the primer product was improved. This step was performed for each primer pair independently and the sizes of the individual amplification products were defined. Each 30 µL of reaction mixture contained 15 µL RedTaq^®^ ReadyMix™ PCR Reaction Mix (Sigma-Aldrich, St. Louis, MO, USA), 0.5 µM of each primer, and 4 ng of soil DNA template. Every PCR was run using an Applied Biosystems Veriti Fast Thermalcycler under the following conditions: preliminary denaturation at 95 °C for 5 min followed by 30 cycles of denaturation at 95 °C for 30 s, primer annealing at 55 °C for 30 s, and extension at 72 °C for 1 min. At the end of the run time, a terminal extension at 72 °C for 15 min was conducted and then immediately maintained at 4 °C. After amplification, 5 µL of each product was used for visualization on agarose gel by electrophoresis (2% agarose, 1 h, 100 V). The sizes of the products were approximately 1000, 900 and 600 bp for bacteria, archaea and fungi, respectively. Subsequently, the simultaneous amplification of bacteria, archaea and fungi products was optimized in a multiplex reaction (multiplex PCR). At the beginning of the process, the multiplex reaction was prepared using the same amount of each primer as for the individual reaction (0.5 µM), but the fungi and archaea amplification products were rather weak in comparison with the products for bacteria. Therefore, in the next amplification reaction, the amount of fungal and archaeal primers was doubled to 1 µM; this approach generated good quality products for each tested group of microorganisms. Both single and multiplex PCR were performed in the same temperature profile and using the same amount of DNA template.

Prior to the digestion of the PCR products with restriction enzyme, the amplicons were purified using a mixture of two enzymes: thermo sensitive alkaline phosphatase and Exonuclease I (Exo-BAP-Mix, EURx) with slight modifications; the purification mixture containing 15 µL of amplicon or an amplicon mixture and 6 µL of reagents were used. The purification process was performed using three different approaches (i) single PCR product (separate reaction for bacteria, fungi and archaea), (ii) pooled PCR products (equal amount of individual amplifications product), and (iii) multiplex PCR products (one reaction for each group of microorganisms: bacteria, archaea, fungi). Thereafter, the process was continued according to the manufacturer’s instructions. Subsequently, the concentrations of purified PCR products were determined spectrophotometrically at 260/280 nm. Purified DNA was used to conduct a digestion reaction. The characteristics of the restriction enzymes used are shown in Table 3.

The PCR products were digested with AluI, Csp6l (Fermentas^®^ International, Burlington, ON, Canada), HaeIII and MspI (EURx) in a 12-µL reaction mixture containing approximately 60 ng of DNA, 0.6 μL of buffer and 0.6 μL of restriction enzyme (10 U/μL). The reaction mixture was incubated at 37 °C for 2 h, subsequently, the reaction was stopped through incubation at 60 °C for 20 min with AluI, Csp6l and MspI, and at 80 °C for 20 min with HaeIII. After the end of the digestion period, 1 μL of product was mixed with 9 μL of deionized formamide and 0.5 μL of DNA fragment length standard (GS-600LIZ, ABI) (Applied Biosystems, Foster City, CA, USA) and placed on a 96-well plate. Fragment size analysis was carried out for a single PCR product with the primers for bacteria, archaea and fungi, pooled PCR products, as well as for multiplex PCR products. The samples were denatured at 94 °C for 3 min and snap-cooled on ice. The fluorescently labelled T-RFs were run through an ABI 3130 xl capillary genetic analyzer (Applied Biosystems, Foster City, CA, USA) in the GeneScan mode and the dye set D. The run parameters were as follows: oven temperature, 60 °C; pre run voltage, 15 kVolts; pre run time, 180 s; injection voltage, 1.6 kVolts: injection time, 15 s; run voltage, 150 kVolts; run time, 1300 s.

The t-RFLP data was analyzed using GeneMaper^®^ Software v 4.0 (Applied Biosystems, Foster City, CA, USA). During the analysis, only peaks with a size range of 50–500 bp were extracted to exclude potential primer peaks and artefacts, and peaks with a signal below 100 relative fluorescence units were discarded from the analysis. The estimation of the relative abundance of the restriction fragments (T-Rf) from the received restriction fragments profile was calculated by dividing the single peak area of the T-Rf by the sum of the area of all of the peaks in the profile. Each peak with an area lower than 1% of the total sum of the area of all peaks was excluded from further analyses. Additionally, the appearance of the restriction fragment was expressed in a binary format (presence, 1, the peak is observed in at least two out of three replicates; absence, 0, if the peak is only observed in one replicate). In a profile obtained from multiplexing and pooling, combination results for various groups of microorganisms were read from different dye channels (G, bacteria; R, archaea; B, fungi). 

The similarity between the different approach methods was calculated based on the restriction fragment pattern [30]. The Jaccard similarity coefficient was calculated according to the formula:

(1) $$J=\frac{N_{AB}}{N_{A}+N_{B}-N_{AB}}$$

*N_AB_*–number of common peaks in two restriction profiles
*N_A_*–number of peaks in restriction profile A
*N_B_*–number of peaks in restriction profile B.

The analyses were performed in triplicate and the data were evaluated using multidimensional scaling to present the relationship between the tested approaches of the techniques. Differences and similarities between t-RFLP approaches were analyzed by principal component analysis (PCA), including the most significant loading of variables. Analysis of variance with the post hoc Tukey HSD test was used to calculate significant differences between tested approaches on different soil types. Pearson’s correlation coefficients were also calculated. All analyses were performed with Statistica Software (version 13. software, StatSoft Inc., Tulsa, OK, USA, 2011). 

## 3. Results

**Figure 1:**
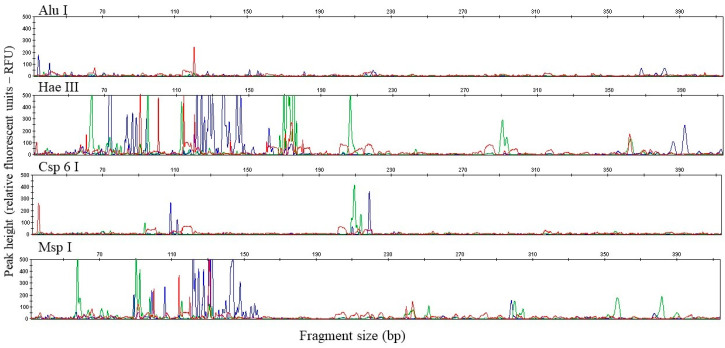
Exemplary electropherograms of T-RFs, the results of multiplex PCR products digestion (obtained for samples from soil 1–S1) with the restriction endonucleases: AluI, HaeIII, Csp6I, MspI. The blue color of peaks represent T-RFs of the PCR products labelled with 6-FAM of the fungal primer. The green and red peaks are the bacteria (HEX) and archaea (ROX), respectively. The T-RF length and height were identified with reference to the internal size standard (LIZ- orange dye) which is not shown in the figures. Explanations: 6-FAM, 6-carboxyfluorescein.

At the beginning of the process, a few endonuclease enzymes were investigated to obtain the best peak pattern (restriction fragment fingerprints). The PCR products amplified by a single, pooling and multiplexing approach were purified and digested with four restriction enzymes: AluI, Csp6l, HaeIII and MspI. Figure 1 represents the exemplary t-RFLP profiles obtained with each enzyme. 

**Figure 2:**
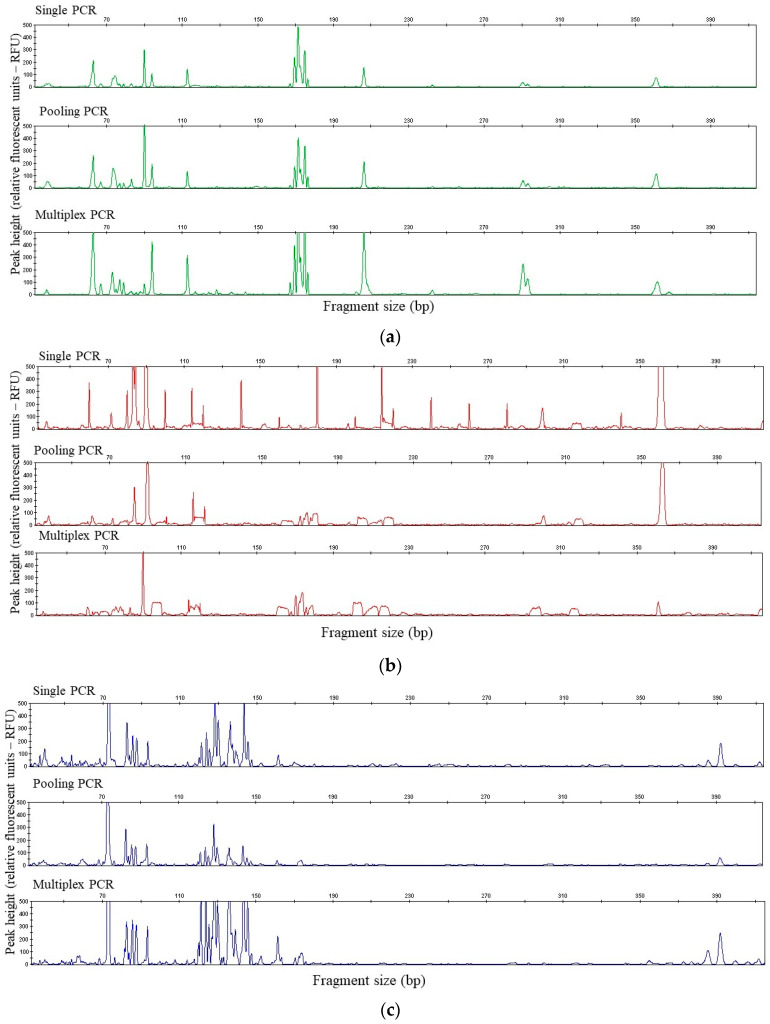
Exemplary electropherograms of the: (**a**) 16S rDNA bacteria, (**b**) 16S rDNA archaea and (**c**) ITS1 fungi T-RFs after digestion with the endonuclease HaeIII.

The results showed that the enzyme AluI did not produce clear peaks of the tested group of microorganisms. On the electropherogram, no peaks for bacteria (green) were visible, only one distinct peak characteristic of archaea ~120 bp (red) and a few minor fungal restriction fragments ~150, 220, 370 bp (blue). For Csp6l endonuclease more peaks were obtained than for AluI. The bacterial restriction profile was characterized by two restriction fragments (~90 and 210 bp), the fungal profile also had two fragments (~110 and 215 bp) and the archaeal profile had three fragments (~90, 115, 200 bp). In contrast, the other enzymes, HaeIII and MspI, produced significantly more restriction fragments. Furthermore, the relative abundance of the peaks obtained was mainly under 200 RFU (relative fluorescence unit), and there were also peaks with off-scale heights. The number of peaks obtained was appropriate and sufficient to characterize the bacterial, archaeal and fungal communities. However, slight differences were found between these two enzymes. The HaeIII enzyme produced a pronounced profile for bacteria (more fragments with a high relative abundance), therefore this enzyme was chosen to make a comparison with the investigated t-RFLP approach. The effectiveness of the multiplex t-RFLP was evaluated by comparing the restriction profile of the multiplex approach with the single and pooling approach. The results from the restriction profiles were obtained for three approaches—single PCR, pooling PCR and multiplex PCR—and are summarized in Figure 2 and in Tables S1 and S2 (Supplementary File). 

As shown in Figure 2a, the t-RFLP profiles obtained for bacteria using the single, pooling and multiplexing techniques were very similar. The observed differences included the relative abundances rather than the size of the fragments obtained. Figure 2b shows a profile of the archaeal communities. A similar profile (for S1 treatment) was obtained using the single and pooling approach. The single t-RFLP approach was characterized by many fragments with a peak height of more than 200 RFU. The most distinctive peaks were 90 and 361 bp, with 53.20% and 28.09% relative abundances, respectively. These fragments were also observed using the pooling and multiplex profile approach. However, their relative abundances were lower, but still significant in each approach. Some peaks were also observed that were only obtained by a single approach electropherogram (100, 339 bp). It is important to note that, of the peaks visible on a single approach PCR electropherogram, many fragments were characterized by a very low area as a result of a low percentage of relative abundance (<1%) and these peaks were discarded from the analyses. This technique was not reported using this scale in other t-RFLP approaches. Many more similarities in the restriction profile were observed between the pooling and multiplex PCR approaches. The results of a comparison between the restriction profiles of the fungi are presented in Figure 2c and Tables S1c and S2c (Supplementary File). The observed profiles were very similar for each tested approach (for S1 as well as for the S2 soil treatment). Characteristic fragments with the same length and a similar height were detected. Only slight differences were found. In Table 4, the similarity index (Jaccard’s coefficient, J) is shown.

The similarities between the approaches considered (single vs. pooling, single vs. multiplex, pooling vs. multiplex) for the bacteria and fungi were close for the two tested soil types (S1 and S2), at over 0.613, excluding the similarity between the single and pooling approach for fungal communities in S2 samples. Nevertheless, a lower number of similarities were found for archaeal communities in S1 and S2 samples (0.208–0.500), although a very high similarity was recorded between single and multiplex approaches in S2 samples.

**Figure 3:**
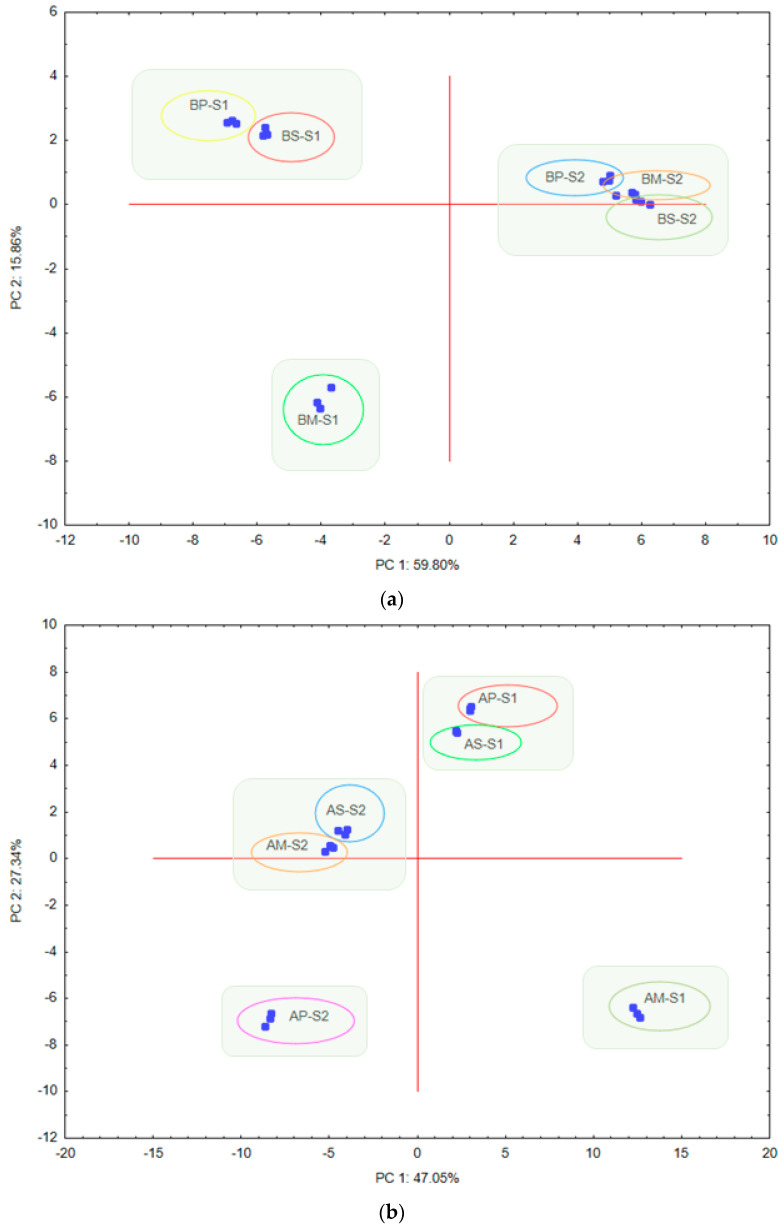

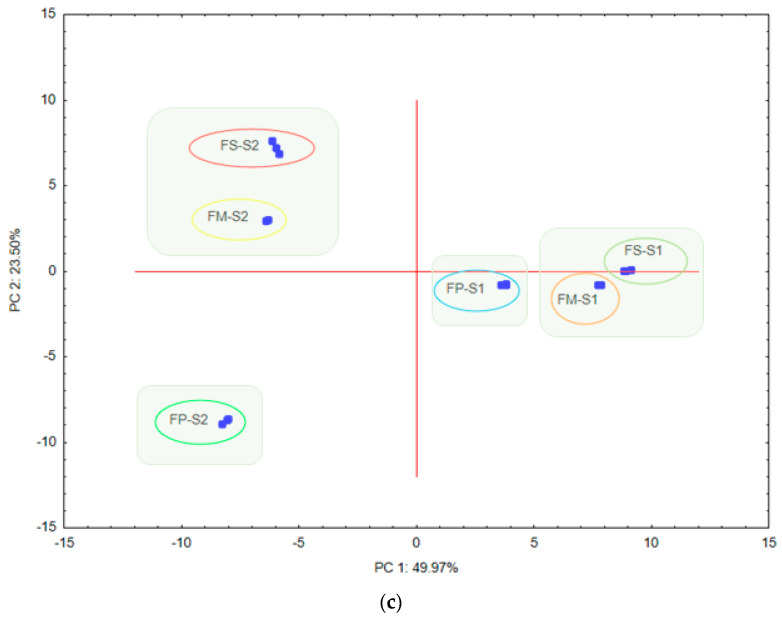
A comparison of the principal components (PC) plots generated with the results of relative abundances and area of peaks of different t-RFLP approaches (single, pooling and multiplex) for S1 and S2 within (**a**) bacterial, (**b**) archaeal and (**c**) fungal communities. Explanations: BS, bacterial single approach; BP, bacterial pooling approach; BM, bacterial multiplex approach; AS, archaeal single approach; AP, archaeal pooling approach; AM, archaeal multiplex approach; FS, fungal single approach; FP, fungal pooling approach; FM, fungal multiplex approach; S1, soil classified as Brunic Arenosol; S2, soil classified as Abruptic Luvisol.

Principal components analysis (PCA) of the three t-RFLP approaches was used to arrange the two types of soil samples (S1 and S2) depending on the HaeIII enzyme used to create the pattern (Figure 3a–c).

PCA grouped the various t-RFLP approach treatments separately with respect to the soil type classified according to the World Reference Base for Soil Resources (WRB) [31] as Brunic Arenosol (S1) and Abruptic Luvisol (S2). Most probably this grouping mainly resulted from the dissimilarities in the soil characteristics (e.g., different nutrient content, soil texture or pH). Moreover, PCA grouped the multiplex and single t-RFLP approach treatments for S2 together, both for archaea and fungi (Figure 3b,c). This result is consistent with the calculated similarity index (Jaccard’s coefficient) (Table 4). Moreover, in Table 5 loadings for the most significant variables (correlation coefficient ≥0.9) along PC1 and PC2 resulting from principal component analysis of relative abundance (RA) and area (AR) of peaks from both soils are presented.

The results confirmed that both the presence of peaks expressed as relative abundance and its quantification based on peak areas were important components of PC1 and PC2 for each microbe group: bacteria, archaea and fungi (Table 5). However, PC2 had the highest values (*R* ≥ 0.9) only in the case of bacteria, indicating negative loadings of relative abundance (RA) and area (AR) for the peak of 63 bp. PC1, which explains 59.80%, 47.05% and 49.97% of the variance for bacteria, archaea and fungi, respectively (Figure 3), had the highest positive loadings (*R* ≥ 0.9) for relative abundance of peak sizes 77, 94, 113, 170, 209, and 360 bp, and for area of peaks with 360 bp for bacteria, 298 bp of both RA and AR for archaea, and 85, 88, 125, 128, 143, 145, and 445 bp of RA and 83, 88, 93, 121, 123, and 125 bp of AR for fungi (Table 5).

**Figure 4:**
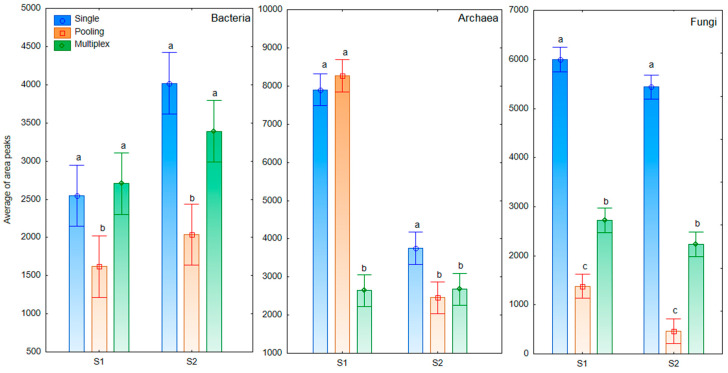
Average of area peaks calculated for proposed method approach for S1 and S2 and each microbial group. Explanation: S1, soil classified as Brunic Arenosol; S2, soil classified as Abruptic Luvisol; small letters on each bar mean significant differences (results of the Tukey’s HSD test at 0.05 level).

Significant differences between proposed approaches of t-RFLP were estimated by the post hoc Tukey HSD test at the *p* < 0.05 significance level and results are presented in Figure 4.

The results indicated that although the significant differences were found in the values of average area of peaks between tested approaches of t-RFLP (Figure 4), the number of peaks in all tested assays was similar for bacteria and fungi, and significantly higher in the multiplexing approach in comparison to multiplexing for archaea (Tables S1 and S2). The results showed that use of the specific t-RFLP approach was dependent on the soil type. Pearson correlation coefficients indicated significant correlations (*p* < 0.05) between single, pooling and multiplexing approaches for bacterial and fungal results (−0.863 and −0.846) on S2 soil and between single and pooling approach for archaea and fungi (0.957) on S1 soil.

## 4. Discussion

The presented study was performed in order to optimize the best conditions for the multiplex t-RFLP method, which consists of multiplex PCR and t-RFLP. This combination of multiplexing techniques presented opportunities to study the soil microbial diversity and structure using a multi-taxonomic approach. The results presented show that the t-RFLP method with a multiplex variation could be an effective and reliable tool for describing the genetic diversity of various microbiological taxonomic groups in agricultural soil samples.

T-RFLP is a useful tool in environmental microbiology for assessing the diversity of microbial populations and shifts in communities. Furthermore, this method is widely used in analysis, especially for soil samples [29]. One of the common matrix genes is bacterial 16S rDNA [32,33], but other genes may also be used for this technique: fungal ribosomal genes [34,35], archaeal 16S rDNA genes [36,37], and functional genes [38,39]. T-RFLP fingerprinting is a pattern of t-RFs, the composite of DNA restriction fragments with characteristic lengths. The multiplex PCR approach allows for the simultaneous amplification of genes from different microbial groups in one reaction and this is a significant advance in relation to t-RFLP.

The use of a variety of fluorescently labelled primers presents the opportunity for fragment separation from various DNA matrices. Therefore, different microbial populations are represented by various fluorescent dyes. The selection of the fluorescent dyes should be based on the emission spectrum (selected dyes should emit different wavelengths). In the presented study, we selected three quite common and accessible fluorescent dyes—6-FAM, ROX and HEX—which were characterized by different emission spectra and intensities, thus making results easy to detect.

The single PCR-t-RFLP method could be replaced by a multi-taxonomic approach, the development of which was initiated by Singh et al. [14]. The authors analyzed microbial communities using primers specific for rhizobia/agrobacteria, fungi and eubacteria. This work indicates that t-RFLP in multiplex modifications is as reproducible and consistent as t-RFLP, which was tested in replication using two different soil types. Our presented work proves that the multiplex t-RFLP tool, including pooling and multiplexing approaches, is a viable alternative for analyses of different microbial taxons as opposed to single t-RFLP. However, different approaches (pooling or multiplexing) gave various effects on sandy and silty soils, which should be taken under consideration at the beginning of new study. Grouping of different approaches within various soil types (Brunic Arenosol, Abruptic Luvisol) [31] mainly resulted from the dissimilarities in the soil parameters (physicochemical and biological properties, grain size, elemental composition) [40]. The results obtained for bacteria and fungi show that the restriction profiles of this group of microorganisms from agricultural soil in both the single and multiplexing approach are very similar. However, for archaea the best results were obtained for the pooling approach. Although some differences were detected, major differences were observed in peaks that were below the applied threshold, with relative abundances lower than 1%. This also applies to some observed differences between Jaccard’s coefficient index, which could be the effect of the presence of peaks under the threshold (i.e., under 1% relative abundance). In the analysis of the results, this type of peak was excluded from further consideration (they were assumed to be artefacts, pollution or non-specific products) [41,42].

Although there were advantages to this approach, there were also several methodological limitations, which should be considered during implementation. The multiplex t-RFLP approach has the same limitations as t-RFLP or other molecular, PCR based techniques [43,44]. The most important and most common of these limitations are discussed below. Firstly, the parameters of DNA amplification could be optimized with non-fluorescent primers, but the differences between the electrophoretic mobility of fluorescent and non-fluorescent amplicons should be noted. Fluorescently labelled DNA may have a different electrophoretic mobility [45]. The second issue was the ability of the restriction endonucleases to resolve a unique fragment pattern. Usually, four base-pair recognition site enzymes are useful in t-RFLP, but the choice of enzyme with the best resolution should be evaluated empirically in the analyzed samples. Next, the detection of restriction fragments is more precise when a capillary sequencer analyzer is used [46]. However, the capillary system is based on electrokinetic sample injection using the charge of the molecule to inject the samples into the capillaries [46]. Due to this, the digestion reaction mixture should be purified to remove smaller molecules, salts and PCR primers [47,48], although this step may cause a decrease in the concentration of DNA. This consideration of the most important issues of the process demonstrates the need for the optimization and improvement of the parameters of the reaction.

### 4.1. Improvement of the Parameters of the Reaction

We investigated the possibility of using the pooling and multiplexing approaches of t-RFLP in comparison to a singleplex assay as efficient biomarkers of microbial diversity of agricultural soil including bacterial, archaeal and fungal communities. It is worth noting that the effectiveness of pooling and multiplexing method could be also related to soil characteristics. Therefore, before the study of many samples, we recommend checking the quality of the results from both proposed approaches (pooling and multiplexing) in comparison to a single assay using a few selected samples of each soil type. Based on the obtained results it was observed that the multiplexing approach was more appropriate for sandy soil (S1), while both assay multiplexing and pooling work equally well for silty soil (S2). Moreover, on the basis of the presented results it might be suggested that for analyses of two microbial groups (bacteria and fungi or bacteria and archaea) that the pooling approach gave better results, while multiplexing t-RFLP could be recommended as an effective approach to study archaea and fungi in the same reaction. However, the potential of the multiplex t-RFLP approach for the study of community diversity and structure could be used and developed for various habitats: sediments, contaminated soil, sewage sludge or organic wastes. Furthermore, the multiplex t-RFLP approach in addition to the 16S rDNA and ITS1 gene could also include genes coding a variety of functional groups. Numerous methodological perspectives can result in difficulties in receiving the appropriate results, thus in the following brief summary we shed light on these possible problems and their solutions.

#### 4.1.1. DNA Extractions

The method of DNA extraction used determines the quality of the DNA and the possibilities of performing a suitable molecular analysis. A high quality of DNA template is required to obtain a low detection threshold and to achieve the most accurate representation of the soil gene reservoir. The most important problem with the analysis of DNA from soils is the presence of humic acids (the products of organic matter decomposition). These may inhibit *Taq* polymerase and the restriction endonucleases [49]. Humic acid inhibition may be limited by diluting the DNA solution and the use of bovine serum albumin (BSA).

#### 4.1.2. PCR

Two strategies for the PCR approach are possible, single and multiplexing, and both were tested in presented study. The multiplexing approach requires a preliminary check for primer compatibility and the possibility of efficient co-amplification. Another important aspect is that the pair of primers should have similar melting temperatures (Tm) and should not contain large regions of complementarity. Multiplexing-pooling strategies (pooling samples after a single PCR) are generally easier to implement due to the different efficiencies of primers, and these were also included in the presented research. The amplification step of multiplex PCR may be difficult to perform due to the equal number of amplicons for each tested group, particularly since that effectiveness depends on the fluorescent dye used. In pooling strategies, this inconvenience may be eliminated by adding different amounts of each individually amplified PCR product, which allows for the achievement of similar peak intensities. In both strategies, it is necessary to use different fluorescent dyes to label the primers. In selecting dyes for analysis, one should consider spectral calibration reagents for the dye sets. Furthermore, when a fluorescent dye is assigned to PCR products, the most intense dyes should be matched with PCR products with a low recovery rate; it follows that less intense dyes should be used with products with a good recovery rate [50]. In Table 6, helpful information about the emission spectra and the intensities of a selection of fluorescent dyes [51] are presented; these parameters are needed for selection and matching of fluorescent dyes.

#### 4.1.3. Restriction Digestion

Concentrations of DNA are closely connected with the amount of endonuclease. Digestion performed with a high concentration of template will result in many high peaks, thereby preventing a meaningful comparison between the size of the peaks corresponding to the standards. It follows that digestion with a very low amount of DNA produced many low height peaks, which were difficult to interpret. In addition, loss of restriction enzyme cut site specificity could cause nonspecifically restriction referred to as “star activity” [52]. The selection of restriction endonucleases could be supported with the use of bioinformatic programs such MiCA [53], TRiFLe [23] and the ARB implemented tool TRF-CUT [54].

#### 4.1.4. Optimizing Capillary Electrophoresis with 3130 Genetic Analyzer Instruments (Applied Biosystems)

The recommended reaction mixture is the following: 0.5 µL of biological sample, 0.5 µL of internal size standard and 9.0 µL of Hi-Di^TM^ formamide. However, if the signal obtained from the sample is very high then dilutions should be prepared. Another solution for an overly high intensity of sample peak is a decrease in the injection time and/or injection voltage. In turn, if the signal from the sample is too weak then the injection time or voltage should be increased. The obtained intensity of the internal size of the standard peaks should be between 30 and 100% of the sample peak intensity [55].

## 5. Conclusions

T-RFLP as a molecular technique for the evaluation and characterization of microbial communities and is a well-described tool. However, the pooling and multiplexing t-RFLP approaches for the detection of bacteria, archaea and fungi in one reaction, which were tested and described in this paper, are not common. The use of pooling and multiplexing approaches allows better understanding of the interactions between the different taxons and microbe groups. It is also relevant to interpret the relationship between the soil microorganisms (bacteria, fungi and archaea), their structural and functional diversity, and soil properties. On the other hand, attempts to evaluate microbial communities relying only on genetic parameters should be performed with great caution. Thus, this molecular study of genetic soil microbial diversity should be complemented with the results from other fingerprinting methods, such as DGGE, and also those from metabolic status measurements, such as enzymatic activity or the community level physiological profiling (CLPP) method [56]. Such a combination of analyses will certainly provide multilevel information about microbiological soil status.

The presented paper shows the optimization stages of the developed multiplex t-RFLP method used to analyze bacterial, archaeal and fungal communities in soil samples. It also describes the most important points that are crucial for this type of analysis.

The results obtained using the developed multiplex t-RFLP method, including multiplexing and pooling approaches, differed slightly for the studied soils, which might be connected with various physicochemical properties of the tested soils. Significant differences between pooling and multiplexing approaches were observed for various microbial groups in sandy and silty soils. The multiplexing approach was more appropriate for sandy soil, while both assay multiplexing and pooling were suitable for silty soil. Moreover, for analyses of two microbial groups (bacteria and fungi or bacteria and archaea) the pooling approach yielded better results, while multiplexing t-RFLP can be recommended as an effective approach to study archaea and fungi.

Although this approach has drawbacks and limitations related to DNA quality and presence of inhibitors, and requires optimization for each type of soil, we present it as reliable, rapid and cost-effective tool for soil microbial community analysis.

## Figures and Tables

**Table 1:** Primers used for single and multiplex PCR.

Primer	Sequences (5′-3′)	Length (bases)	Tm ^a^ (°C)	GC (%)	Fluorescent Label	Specificity	References
Ar3 f	TTC CGG TTG ATC CTG CCG GA	20	55.9	60	-	Archaea	[24]
Ar9 ^c^ r	CCC GCC AAT TCC TTT AAG TTT C	22	60.0	45	ROX (red)	Archaea	[25]
63 f	AGG CCT AAC ACA TGC AAG TC	20	51.8	50	-	Eubacteria	[26]
1087 ^c^ r	CTC GTT GCG GGA CTT ACC CC	20	57.9	65	HEX (green)	Eubacteria	[27]
ITS1 f	CTT GGT CAT TTA GAG GAA GTA A	22	49.2	36	6-FAM ^b^ (blue)	Fungi	[28]
ITS4 r	TCC GCT TAT TGA TAT GC	20	49.7	45	-	Fungi	[29]

^a^ Tm, melting temperature of primers. ^b^ 6-FAM, 6-carboxyfluorescein. ^c^ in modifications (fluorescent labelling).

**Table 2:** Outline of the Terminal Restriction Fragment Length Polymorphism (t-RFLP) analysis procedure.

t-RFLP Approach	PCR *	Restriction Digestion **	Fragment Analysis
Single	3 individual reactions	3 individual digestions for each enzyme	Individual
Pooling	3 individual reactions	1 digestion with mixture of individual PCR products for each enzyme	Mixture
Multiplexing	1 multiplex reaction	1 digestion of multiplex PCR products	Mixture

* PCR products were obtained with the same three pairs of primers. ** Restriction digestion products were obtained with the same restriction enzymes.

**Table 3:** Characteristics of the restriction enzymes used in the t-RFLP approach.

Restriction Enzyme	Recognized Sequences of the Restriction Enzyme	Condition of Restriction	Condition of Inactivation Enzyme	Enzyme Running Buffer- Composition
AluI	5′ AG↓CT 3′ 3′ TC↑GA 5′	37 °C for 60 min	60 °C for 20 min	10× Buffer Tango
Csp6I	5′ G↓TAC 3′ 3′ CAT↑G 5′	37 °C for 60 min	60 °C for 20 min	10× Buffer B
HaeIII	5′ GG↓CC 3′ 3′ CC↑GG 5′	37 °C for 60 min	80 °C for 20 min	Buffer 1× with BSA
MspI	5′ C↓CGG 3′ 3′ GGC↑C 5′	37 °C for 60 min	60 °C for 20 min	1× ONE Buffer

↑,↓—Indicates the point of the cuts of the DNA strain by enzyme.

**Table 4:** Jaccard’s coefficient (J) * between the restriction profile obtained for various t-RFLP approaches.

Tested Soil	TRFLP Approach	Bacteria	Archaea	Fungi
Soil 1 (S1)	Single vs. pooling	0.842	0.333	0.750
	Single vs. multiplex	0.823	0.208	0.850
	Pooling vs. multiplex	0.789	0.208	0.714
Soil 2 (S2)	Single vs. pooling	0.750	0.433	0.469
	Single vs. multiplex	0.818	0.905	0.773
	Pooling vs. multiplex	0.909	0.500	0.613

* Jaccard’s coefficient was calculated based on number of peaks; the peak was taken into account when was observed in at least two out of three replicates; no differences in the number of peaks between replicates were observed.

**Table 5:** Loadings for the most significant variable along PC1 and PC2 resulting from principal components analysis for bacteria, archaea and fungi (*R* ≥ 0.9). RA, relative abundance and AR, area of peaks. 63–361, size of peaks in base pairs.

Bacteria			Archaea		Fungi	
Variable	PC1	PC2	Variable	PC1	Variable	PC1
63-RA		−0.981	298-RA	0.948	85-RA	0.931
67-RA	−0.983		298-AR	0.960	86-RA	−0.952
74-RA	−0.957				88-RA	0.922
77-RA	0.975				125-RA	0.938
93-RA	−0.975				128-RA	0.979
94-RA	0.980				140-RA	−0.913
113-RA	0.939				143-RA	0.962
170-RA	0.930				145-RA	0.904
171-RA	−0.949				198-RA	−0.943
173-RA	−0.965				445-RA	0.991
206-RA	−0.997				478-RA	−0.911
209-RA	0,986				544-RA	−0.926
290-RA	−0.993				83-AR	0.919
360-RA	0.981				88-AR	0.941
361-RA	−0.937				93-AR	0.991
63-AR		−0.971			121-AR	0.920
67-AR	−0.968				123-AR	0.911
74-AR	−0.956				125-AR	0.970
93-AR	−0.956					
173-AR	−0.924					
290-AR	−0.988					
360-AR	0.960					
361-AR	−0.931					

**Table 6:** Emission spectra of fluorescent dye.

Dye	Emission Max (Wavelength nm)	Intensity (not Scaled)
6- FAM	520	****
VIC	550	****
NED	570	**
PET	590	*
LIZ	655	***
HEX	556	***
ROX	600	*

Explanations: **** the strongest fluorescent signal, * the weakest fluorescent signal, 6-FAM and VIC dye emits the strongest fluorescent signal, and PET and ROX emits the weakest signal.

## Supplementary Materials

The following are available online at https://www.mdpi.com/1424-8220/20/11/3292/s1, Table S1: T-RFs detected in a treatment of soil classified as Brunic Arenosol (S1) DNA sample after HaeIII digestion of the (**a**) 16S rDNA bacteria, (**b**) 16S rDNA archaea, (**c**) ITS1 fungi PCR products. Number “1” represents the presence of a fragment and “0” means absence. The relative abundance is the percentage of each fragment calculated based on the total area. The value after ± means standard deviation. Table S2: T-RFs detected in a treatment of soil classified as Abruptic Luvisol (S2) DNA sample after HaeIII digestion of the (**a**) 16S rDNA bacteria, (**b**) 16S rDNA archaea, (**c**) ITS1 fungi PCR products. Number “1” represents the presence of a fragment and “0” means absence. The relative abundance is the percentage of each fragment calculated based on the total area. The value after ± means standard deviation.
