# Supplementary material for: Methodological Aspects of Multiplex Terminal Restriction Fragment Length Polymorphism-Technique to Describe the Genetic Diversity of Soil Bacteria, Archaea and Fungi
Source: Sensors (Basel). 2020 Jun 9;20(11):3292. doi: 10.3390/s20113292 (PMC7309186; doi:10.3390/s20113292)
Supplement: Supplementary file 1 [file sensors-20-03292-s001.pdf]

Supplementary File

Table S1. T-RFs detected in a treatment of soil classified as *Brunic Arenosal* (S1) DNA sample after *HaeIII* digestion of the (**a**) 16S rDNA bacteria, (**b**) 16S rDNA archaea, (**c**) ITS 1fungi PCR products. The number "1" represents the presence of a fragment and the "0" means absence. Relative abundance is the percentage of each fragment calculated based on total area. Value after ± means standard deviation.

Fragment size (bp)	Bacteria					
	**Single PCR**	**Relative abundance (%)**	**Pooling PCR**	**Relative abundance (%)**	**Multiplex PCR**	**Relative abundance (%)**
63	1	9.71±0.12	1	9.55±0.12	1	35.14±0.07
67	1	1.18±0.05	1	1.54±0.09	1	1.27±0.19
74	1	7.77±0.07	1	8.67±0.12	1	3.66±0.10
78	0		1	1.07±0.04	1	1.92±0.14
83	0		1	2.08±0.07	0	
90	1	7.86±0.04	1	13.36±0.26	0	
93	1	3.77±0.36	1	5.18±0.05	1	4.67±0.06
113	1	5.32±0.19	1	3.62±0.14	1	3.37±0.09
170	1	7.74±0.38	1	4.17±0.10	1	3.81±0.09
171	1	18.99±0.07	1	12.43±0.21	1	11.26±0.08
173	1	6.08±0.09	1	4.67±0.08	1	3.38±0.04
175	1	11.24±0.55	1	10.56±0.07	1	7.33±0.05
177	1	1.35±0.21	1	1.62±0.10	1	10.67±0.22
206	1	6.83±0.32	1	7.58±0.21	1	5.89±0.21
290	1	2.64±0.23	1	3.29±0.09	1	2.35±0.07
293	1	1.43±0.11	1	1.69±0.03	1	2.90±0.07
361	1	6.23±0.31	1	6.58±0.08	1	2.37±0.05
440	1	1.87±0.04	1	1.06±0.04	0	
446	0		1	1.29±0.06	0	
Total fragments	16		19		15	

(**a**)

Fragment size (bp)	Archaea					
	**Single PCR**	**Relative abundance (%)**	**Pooling PCR**	**Relative abundance (%)**	**Multiplex PCR**	**Relative abundance (%)**
60	1	2.61±0.07	0		1	1.24±0.07
70	0		0		1	1.46±0.04
75	0		0		1	2.82±0.07
78	0		0		1	1.84±0.05
83	1	9.51±0.06	1	7.51±0.04	0	
90	1	53.20±0.46	1	39.37±0.54	1	7.41±0.03
96	0		1	1.06±0.08	1	2.92±0.07
98	0		0		1	5.82±0.09
100	1	1.47±0.06	0		0	
114	1	1.52±0.08	0		1	6.93±0.04
118	0		0		1	3.65±0.05
149	0		1	1.02±0.04	0	
162	0		0		1	6.31±0.07
165	0		0		1	2.87±0.05
172	0		0		1	4.19±0.07
174	0		1	1.29±0.07	1	5.48±0.16
179	0		0		1	4.13±0.06

202	0		0		1	10.31±0.07
209	0		0		1	6.06±0.06
212	0		0		1	7.15±0.07
298	1	1.34±0.03	1	1.93±0.08	1	6.18±0.03
304	0		1	1.33±0.10	0	
317	0		0		1	7.38±0.04
339	1	2.27±0.12	0		0	
361	1	28.09±0.38	1	46.49±0.29	1	2.61±0.05
415	0		0		1	3.25±0.05
Total fragments	8		8		21	

(**b**)

**Fragment size**	**Fungi**					
	**Single PCR**	**Relative abundance (%)**	**Pooling PCR**	**Relative abundance (%)**	**Multiplex PCR**	**Relative abundance (%)**
60	0		1	3.15±0.06	0	
73	1	20.85±0.03	1	35.65±0.41	1	16.67±0.33
83	1	3.19±0.02	1	6.81±0.14	1	3.07±0.06
85	1	3.13±0.07	1	2.74±0.06	1	1.87±0.07
88	1	1.96±0.06	1	2.68±0.03	1	2.85±0.06
93	1	1.43±0.06	1	3.75±0.06	1	2.54±0.03
121	1	1.43±0.07	1	1.45±0.06	1	2.94±0.06
123	1	1.85±0.08	1	1.93±0.05	1	4.35±0.09
125	1	1.20±0.04	1	2.23±0.04	1	1.96±0.02
128	1	6.77±0.04	1	7.27±0.05	1	7.73±0.23
130	1	5.92±0.05	1	3.15±0.06	1	3.68±0.07
137	1	6.21±0.04	1	1.09±0.05	1	2.77±0.04
139	1	2.86±0.06	0		1	3.21±0.06
143	1	6.93±0.05	1	3.62±0.17	1	6.85±0.04
145	1	1.86±0.05	1	1.05±0.05	1	3.14±0.06
161	0		0		1	3.64±0.05
173	0		1	1.77±0.09	0	
385	0		0		1	2.63±0.05
392	1	3.14±0.14	1	2.25±0.08	1	8.15±0.06
445	1	25.59±0.16	1	19.41±0.85	1	20.40±0.14
453	1	1.66±0.14	0		1	1.56±0.04
528	1	4.02±0.05	0		0	
Total fragments	18		17		19	

(**c**)

Table S2. T-RFs detected in a treatment of soil classified as *Abruptic Luvisol* (S2) DNA sample after *HaeIII* digestion of the (**a**) 16S rDNA bacteria, (**b**) 16S rDNA archaea, (**c**) ITS1 fungi PCR products. The number "1" represents the presence of a fragment and the "0" means absence. Relative abundance is the percentage of each fragment calculated based on total area. Value after ± means standard deviation.

Fragment size (bp)	Bacteria					
	Single PCR	Relative abundance (%)	Pooling PCR	Relative abundance (%)	Multiplex PCR	Relative abundance (%)
63	1	18.26±0.68	1	18.74±0.37	1	16.73±0.31
77	1	5.08±1.05	1	4.15±0.15	1	5.51±0.33
89	0		1	6.55±0.36	0	
94	1	7.86±0.04	1	2.99±0.37	1	3.70±0.31
113	1	17.42±0.32	1	17.58±0.61	1	12.98±0.36
117	0		1	2.73±0.31	0	1.59±0.10
170	1	24.00±0.70	1	21.46±1.06	1	33.98±1.75
175	1	14.06±0.51	1	11.87±0.55	1	10.36±0.46
203	1	2.57±0.07	0		0	
209	1	9.11±0.12	1	8.05±0.17	1	8.44±0.46
293	1	3.46±0.17	1	2.69±0.26	1	3.42±0.27
360	1	2.84±0.03	1	3.17±0.23	1	3.30±0.15
Total fragments	10		11		10	

(**a**)

Fragment size (bp)	Archaea					
	Single PCR	Relative abundance (%)	Pooling PCR	Relative abundance (%)	Multiplex PCR	Relative abundance (%)
60	0		1	2,24±0,09	0	
83	1	1,57±0,23	0		1	2,22±0,12
89	1	48,68±2,27	1	22,21±2,06	1	59,16±2,22
100	0		1	2,91±0,3	0	
114	1	1,59±0,21	1	3,44±0,29	1	1,91±0,14
120	1	1,61±0,33	1	2,18±0,09	1	1,89±0,13
164	0		1	2,33±0,11	1	1,51±0,13
173	0		1	2,51±0,26	0	
180	1	1,69±0,25	1	3,53±0,12	1	2,01±0,06
200	0		1	2,06±0,07	0	
207	0		1	2,54±0,49	0	
212	0		1	2,33±0,09	0	
214	1	2,16±0,99	0		1	2,28±0,15
219	1	1,63±0,38	1	1,99±0,07	1	2,03±0,23
240	1	1,91±0,35	1	4,36±0,29	1	2,81±0,08
260	0		1	2,33±0,04	1	1,7±0,19
280	1	1,52±0,22	1	3,24±0,23	1	1,83±0,07
300	1	2,03±0,16	1	4,21±0,19	1	2,36±0,08
315	1	1,19±0,17	0		1	1,49±0,17
319	1	1,55±0,2	0		1	1,91±0,16
340	1	1,6±0,23	1	3,43±0,19	1	1,96±0,02
360	1	23,2±1,74	1	12,99±1,13	1	2,98±0,16
400	0		1	2,65±0,16	0	
414	1	1,74±0,48	0		1	2,18±0,12
418	1	1,89±0,4	0		1	2,13±0,32
439	0		1	2,85±0,17	1	

458	1	1,61±0,24	1	3,51±0,2	1	1,92±0,11
477	0		1	3,23±0,09	0	
510	1	1,19±0,13	1	2,18±0,3	1	1,78±0,06
514	1	1,59±0,25	1	4,75±0,26	1	1,92±0,29
Total fragments	18		23		20	

(**b**)

**Fragment size (bp)**	**Fungi**					
	**Single PCR**	**Relative abundance (%)**	**Pooling PCR**	**Relative abundance (%)**	**Multiplex PCR**	**Relative abundance (%)**
73	1	11,64±0,17	1	10,14±0,77	1	21,78±0,17
82	1	9,7±0,19	1	5,04±3,21	1	19,75±0,06
86	1	3,07±0,04	1	3,09±0,27	1	3,87±0,08
101	0		1	1,21±0,16	0	
110	0		1	1,46±0,13	0	
118	0		1	1,95±0,83	1	0,44±0,02
124	1	2,6±0,01	1	1,82±0,12	1	1,77±0,05
130	1	4,55±0,01	1	2,69±0,24	1	5,01±0,29
140	1	1,88±0,04	1	1,93±0,16	1	7,08±0,22
146	1	6,01±0,04	1	4,72±0,32	1	2,39±0,25
153	1	4,42±0,07	1	3,14±0,101	1	2,99±0,09
171	0		1	3,17±0,29	0	
180	0		1	3,49±0,91	0	
198	0		1	1,58±0,15	1	0,42±0,02
240	1	1,96±0,01	1	1,75±0,39	1	2,24±0,04
295	0		1	1,34±0,15	0	
299	0		1	3,05±0,28	1	0,72±0,02
340	0		1	1,28±0,12	0	
372	0		1	1,86±0,17	1	0,49±0,01
392	0		1	8,37±0,79	0	
433	1	1,95±0,04	0		1	1,88±0,26
439	1	2,57±0,05	1	1,68±0,15	1	5,11±0,26
441	0		1	1,27±0,12	0	
448	1	1,72±0,13	0		1	3,24±0,04
457	1	23±0,02	1	16,17±0,53	1	5,69±0,24
462/463	1	4,4±0,1	1	2,26±0,36	1	7,02±0,17
477/478	1	9,36±0,067	1	6,33±0,89	1	2,23±0,07
514	1	1,47±0,1	1	2,06±0,19	1	2,81±0,05
540	0		1	1,54±0,47	0	
544	0		1	2,66±0,39	0	
563	1	4,26±0,1	1	2,98±0,38	1	3,05±0,09
Total fragments	18		29		21	

(**c**)
